# Therapeutic Potential of a Vasopressin V2 Receptor Antagonist for Calcium Channel Blocker-Associated Edema with Vasospastic Angina

**DOI:** 10.1155/2022/9550006

**Published:** 2022-07-08

**Authors:** Kojiro Toda, Masashi Fujino, Kota Murai, Teruo Noguchi

**Affiliations:** Department of Cardiovascular Medicine, National Cerebral and Cardiovascular Centre, Suita, Japan

## Abstract

Calcium channel blocker- (CCB-) associated peripheral edema does not resolve without CCB discontinuation or dose reduction. However, renin–angiotensin system (RAS) inhibitors have been reported to be effective for CCB-associated edema. We report a case of vasospastic angina with refractory CCB-associated edema. A 78-year-old man had refractory edema induced by a CCB. It was successfully treated with tolvaptan, an active vasopressin V2 receptor antagonist. The aim of this case report is to understand the mechanism and treatment of CCB-associated peripheral edema and how tolvaptan affects peripheral edema.

## 1. Introduction

Calcium channel blockers (CCBs) are commonly used antihypertensive drugs. Peripheral edema is a common dose-dependent adverse effect. CCB-associated peripheral edema is thought to result from preferential arteriolar dilatation. Renin–angiotensin system (RAS) inhibitors can reduce CCB-associated peripheral edema by decreasing postcapillary resistance, which normalizes intracapillary pressure and reduces fluid extravasation. No other medications for CCB-associated peripheral edema have been reported. We describe successful treatment of CCB-associated peripheral edema using tolvaptan, an active vasopressin V2 receptor antagonist, in a patient with vasospastic angina who had been resistant to conventional diuretic and RAS inhibitor therapy.

## 2. Case Presentation

**Figure 1:**
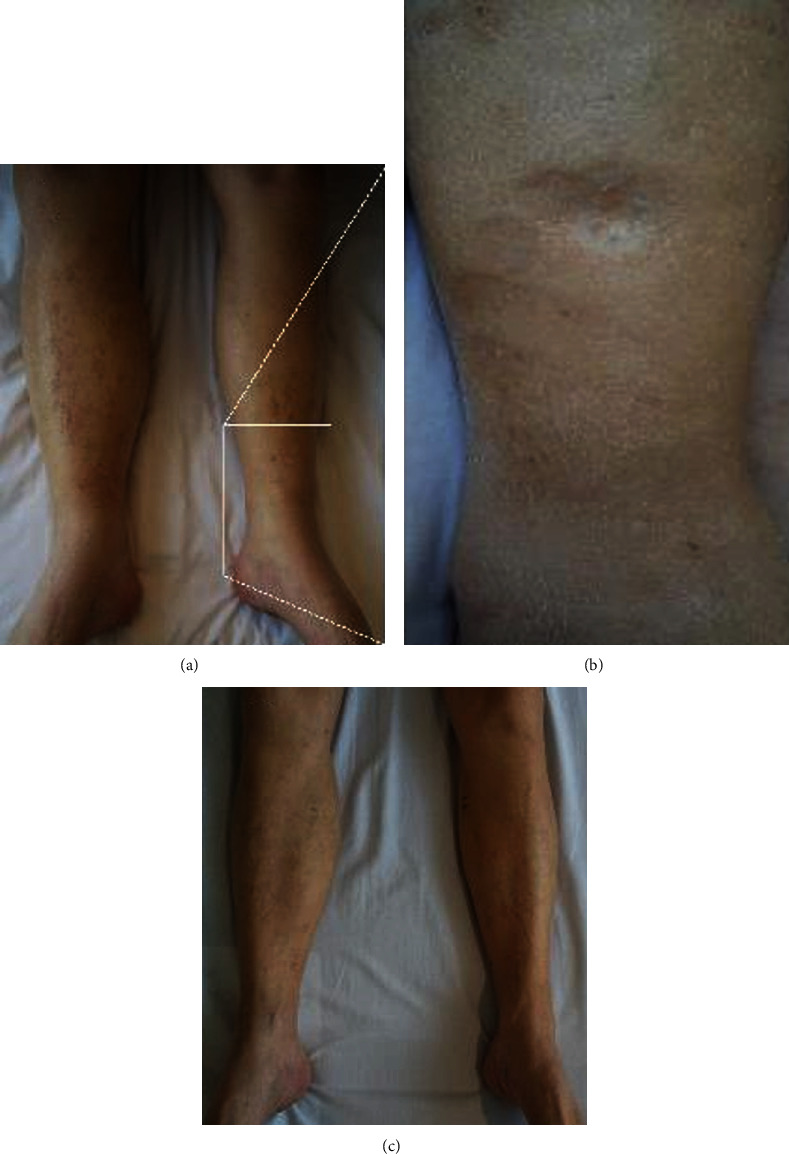
Photographs of the lower extremities. (a) Severe edema of the lower extremities. (b) Pitting edema in a magnified area of (a). (c) Tolvaptan reduced edema without side effects, leading to a body weight decrease of 12 kg.

A 78-year-old man was admitted to our hospital with bilateral lower extremity swelling. He could not put on shoes of his usual size due to severe edema. His blood pressure was 126/62 mmHg, and pulse was 83 beats/min. Compared to his usual body weight, there was a 10 kg increase, up to 71 kg. Physical examination was notable for pitting edema on both lower extremities (Figures 1(a) and 1(b)). Furthermore, he had edema on his face and upper extremities. His jugular veins were not dilated.

**Figure 2:**
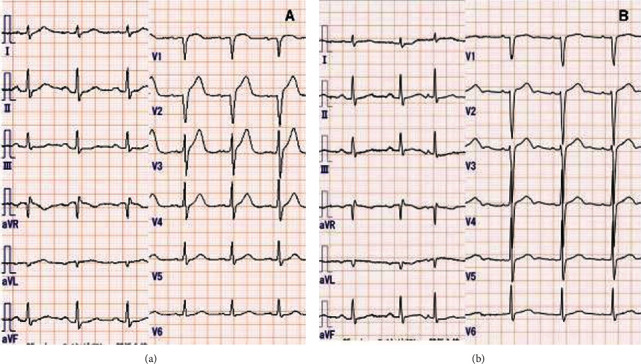
Electrocardiography findings. (a) ST-segment elevation in leads V2–4 during a heart attack. (b) After lingual nitroglycerin therapy, ST-segment elevations resolved.

**Figure 3:**
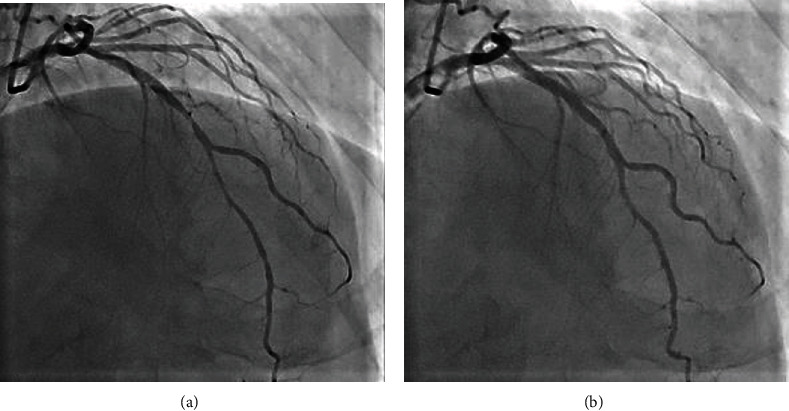
Coronary angiography findings. Left coronary angiography before (a) and after (b) nitroglycerin injection via a catheter, which dilated the stenosis in the middle of the left anterior descending coronary artery.

His medical history included vasospastic angina, chronic kidney disease, and paroxysmal atrial fibrillation. Two months prior, he was diagnosed with vasospastic angina because of transient ST-segment elevation on an electrocardiogram during a heart attack, which was relieved by sublingual nitroglycerin (Figures 2(a) and 2(b)). Coronary angiography showed stenosis of the left anterior descending artery, which improved with nitroglycerin injection into the left coronary artery via a catheter (Figures 3(a) and 3(b)). He was prescribed nifedipine 40 mg daily because he had experienced angina with amlodipine 5 mg per day, but angina with ST-segment elevation recurred several times. Finally, he was discharged after being gradually titrated to nifedipine 80 mg per day, diltiazem 200 mg per day, and nitroglycerin patch 27 mg per 24 hours.

The differential diagnosis included CCB-associated peripheral edema, congestive heart failure, kidney failure, cirrhosis, hypothyroidism, and deep venous thrombosis. Laboratory tests revealed elevated creatinine (1.25 mg/dL) and B-type natriuretic peptide (128 pg/mL; normal range < 18 pg/mL), but no significant changes from baseline. Albumin, liver function, and thyroid hormone levels were within normal limits. The electrocardiogram showed atrial fibrillation with no remarkable ST-segment changes. Chest radiography revealed a cardiothoracic ratio of 48%, but both costophrenic angles were dull. Echocardiography revealed a normal left ventricular ejection fraction of 60%, left ventricular diastolic/systolic diameter of 51/33 mm, interventricular septum/posterior wall thickness of 10/9 mm, mild tricuspid regurgitation, and preserved function of the other cardiac valves. Based on these results, we made a diagnosis of CCB-associated peripheral edema.

As initial treatment, enalapril 2.5 mg per day and furosemide 20 mg per day were administered. The patient could not tolerate a higher dose of enalapril because of dizziness due to hypotension. Furosemide was discontinued because it decreased renal function. After adding spironolactone 25 mg per day, he lost 4 kg of body weight, but edema and pleural effusion remained. Concerned about the recurrence of angina, the patient disagreed with CCB discontinuation or dose reduction. When we tried the active vasopressin V2 receptor antagonist tolvaptan at 7.5 mg per day, his urine output increased and his edema gradually resolved (Figure 1(c)). He lost 12 kg of body weight; his weight decreased to 59 kg. He had no adverse side effects while taking tolvaptan, such as hypernatremia. Renal function remained stable. After taking tolvaptan, laboratory tests revealed serum sodium of 140 mEq/L, creatinine of 1.21 mg/dL, and B-type natriuretic peptide of 81.0 pg/mL. After discharge, the frequency of tolvaptan was decreased to every other day, with no recurrences of peripheral edema and no side effects from tolvaptan.

## 3. Discussion

This case indicated that tolvaptan has favorable effects on CCB-associated peripheral edema. Peripheral edema is a common adverse effect of CCBs. The incidence of peripheral edema is both dose- and duration-dependent. The incidence might exceed 80% with very high doses of dihydropyridine CCBs [1]. Furthermore, conventional racemic amlodipine causes edema more commonly than the S-isomer of amlodipine [2]. Treatment of CCB-associated peripheral edema consists of CCB dose reduction or discontinuation. A previous meta-analysis reported that combining angiotensin-converting enzyme (ACE) inhibitors or angiotensin II receptor blockers (ARBs) with CCBs reduces the incidence of CCB-associated edema by 38%. The CCB discontinuation rate due to peripheral edema with RAS inhibitors was 62% lower than with CCB monotherapy [3]. In addition, ACE inhibitors seem to be more effective than ARBs in reducing CCB-associated peripheral edema [3]. However, edema is sometimes refractory. Peripheral edema is considered to be the result of peripheral arteriolar dilatation, which increases the pressure gradient between arteriolar and venule capillaries and leads to extravasation of intravascular fluid. RAS inhibitors decrease postcapillary resistance, which normalizes intracapillary pressure and reduces fluid extravasation. This patient required high doses of nifedipine and diltiazem to control angina. The combination of these two drugs increases the blood concentration of nifedipine and potentiates its antihypertensive effects. Thus, he could not tolerate ACE inhibitors at a sufficiently high dose. Dihydropyridine-associated edema occasionally occurs despite the use of RAS inhibitors in addition to a diuretic. Exchanging nifedipine for extended-release diltiazem or felodipine led to patient tolerability and blood pressure control [4]. Although there has been a case report showing that the combination of dihydropyridine and diltiazem lowers blood pressure through their interaction [5], there have been no reports of refractory edema due to that interaction.

This patient was concerned about the recurrence of angina. Thus, he disagreed with CCB discontinuation or dose reduction. Furthermore, since he had mild chronic kidney disease, the edema was extremely severe and his response to conventional diuretics was poor. Refractory peripheral edema developed; low-dose RAS inhibitor therapy did not reduce the edema.

**Figure 4:**
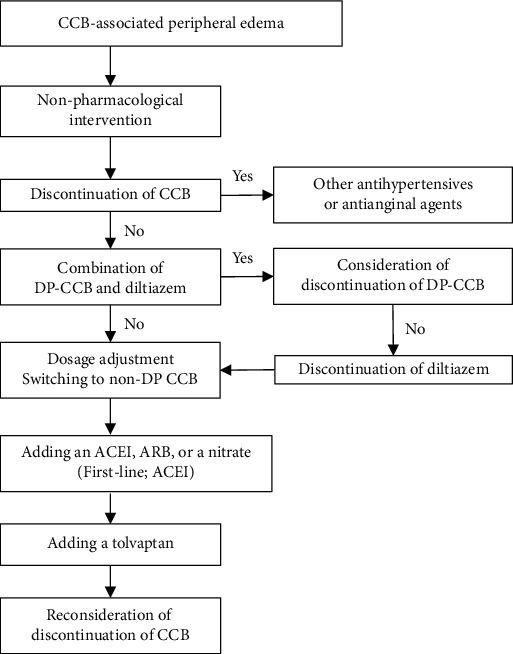
Treatment strategies for patients with CCB-associated peripheral edema. Abbreviations: ACE: angiotensin-converting enzyme; ARB: angiotensin II receptor blocker; CCB: calcium channel blocker; DP: dihydropyridine.

Tolvaptan has a diuretic effect with a mechanism that is different from that of conventional diuretics. Tolvaptan is an orally active vasopressin V2 receptor antagonist that blocks arginine vasopressin from binding to V2 receptors in the distal nephron, which induces the excretion of electrolyte-free water without changing the total level of electrolyte excretion [6]. Although loop diuretics predominantly decrease extracellular water (ECW) rather than intracellular water (ICW), a previous study that involved bioimpedance analysis showed that tolvaptan ameliorates body fluid retention and induces equivalent reductions in ICW and ECW without worsening renal function [7]. Another study showed that tolvaptan restores body fluids by the efficient reduction of intracellular fluid than hemodialysis, by reducing the volume of intracellular fluid by a greater amount and maintaining more fluid in the extracellular space [8]. Tolvaptan induces diuresis of free water and an increase in serum sodium. Because the serum sodium concentration and the interstitial sodium concentration is identical, tolvaptan then induces an increase in the osmolarity of both the intravessel compartment and the interstitial compartment which would accelerate the oncotic forces leading to fluid movement from the intracellular compartment to the interstitial compartment. Tolvaptan helps maintain a lower intravessel volume and promote efficient diuresis, which ultimately decreases the fluid in the interstitium (i.e., edema). To treat CCB-associated peripheral edema, tolvaptan might work by helping fluid move from the intracellular compartment to the interstitial compartment. Furthermore, tolvaptan is reported to be effective for patients with chronic kidney disease [9]. In addition, dihydropyridine CCBs have a sodium diuretic effect; peripheral edema is not due to salt retention [10]. Therefore, classical diuretic treatment is not expected to be effective. However, tolvaptan was effective for this patient with CCB-associated peripheral edema.  Figure 4 shows the strategy for patients with CCB-associated peripheral edema [11].

This case demonstrated that tolvaptan had a favorable effect on CCB-associated peripheral edema. It also suggested that an oral vasopressin V2 receptor antagonist could reduce CCB-associated peripheral edema in a patient with refractory edema even after CCB dose reduction or RAS inhibitor therapy.

## Data Availability

The data used to support the findings of this study are included in the article.

## Abbreviations

ACE: Angiotensin-converting enzyme
ARB: Angiotensin II receptor blocker
CCB: Calcium channel blocker
ECW: Extracellular water
ICW: Intracellular water
RAS: Renin-angiotensin system.
